# Enhanced brightness of bacterial luciferase by bioluminescence resonance energy transfer

**DOI:** 10.1038/s41598-021-94551-4

**Published:** 2021-07-22

**Authors:** Tomomi Kaku, Kazunori Sugiura, Tetsuyuki Entani, Kenji Osabe, Takeharu Nagai

**Affiliations:** SANKEN (The Institute of Scientific and Industrial Research), Osaka University, Ibaraki, Osaka 567-0047 Japan

**Keywords:** Molecular engineering, Biochemistry, Biological techniques, Biophysics, Biotechnology

## Abstract

Using the *lux* operon (*luxCDABE*) of bacterial bioluminescence system as an autonomous luminous reporter has been demonstrated in bacteria, plant and mammalian cells. However, applications of bacterial bioluminescence-based imaging have been limited because of its low brightness. Here, we engineered the bacterial luciferase (heterodimer of luxA and luxB) by fusion with Venus, a bright variant of yellow fluorescent protein, to induce bioluminescence resonance energy transfer (BRET). By using decanal as an externally added substrate, color change and ten-times enhancement of brightness was achieved in *Escherichia coli* when circularly permuted Venus was fused to the C-terminus of luxB. Expression of the Venus-fused luciferase in human embryonic kidney cell lines (HEK293T) or in *Nicotiana benthamiana* leaves together with the substrate biosynthesis-related genes (*luxC*, *luxD* and *luxE*) enhanced the autonomous bioluminescence. We believe the improved luciferase will forge the way towards the potential development of autobioluminescent reporter system allowing spatiotemporal imaging in live cells.

## Introduction

Light production in luminous bacteria results from an enzymatic reaction of a substrate catalyzed by a bacterial luciferase^1–3^. Bacterial luciferase oxidizes reduced flavin mononucleotide (FMNH_2_) and a long-chain fatty aldehyde (RCHO) to yield flavin mononucleotide (FMN) and the corresponding long-chain fatty acid (RCOOH). This reaction concomitantly generates blue-green light with a peak wavelength around 490 nm.

The fundamental enzymes required for bacterial luminescence are encoded by a single operon, *luxCDABE*, which is found in all species of luminous bacteria^2,3^. The *luxA* and *luxB* genes encode for the α and β subunits of a heterodimeric protein of bacterial luciferase, respectively. The *luxC*, *luxD* and *luxE* genes encode for the complex components that serves to synthesize and recirculate fatty aldehyde, which is the substrate for luciferase. Co-expression of the five *lux* genes in non-luminous bacteria or yeast cells shows a light-emitting phenomenon with no external supply of the substrate^4,5^. In mammalian or plant cells, the additional gene expression of FMN oxidoreductase (*luxG*), which provides a sufficient amount of FMNH_2_, together with the five *lux* genes enables stable autobioluminescence^6–9^.

Introduction of genetically encoded reporters into living cells has been widely used to observe biological phenomena. Compared to fluorescence imaging, bioluminescence imaging does not need external excitation illumination that can cause problems such as phototoxicity, photobleaching, and autofluorescence from the specimen. Bacterial luciferase-based reporter is a valuable tool because of its high signal-to-noise ratio and ease of operation. Additionally, a wider spectral information can be obtained from bioluminescence compared to fluorescence because the excitation illumination does not interfere with the spectral analysis. However, the application of bacterial bioluminescence imaging has been limited because of the low brightness^10^. It has been shown that seven-times increment of bacterial bioluminescence allows imaging of single *E. coli* cells with improved spatiotemporal resolution^11^. However, image acquisition using this enhanced luminescence still requires about 10 min of exposure time, which would be difficult to capture biological phenomena that change rapidly. Therefore, a higher luminescence intensity is expected to allow observation of biological phenomena that change within minutes or even less.

To further improve on the signal of the bacterial bioluminescence, we attempted to increase the luminescence of bacterial luciferase by fusion to a fluorescent protein. This approach is based on the phenomenon of bioluminescence resonance energy transfer (BRET) between the luciferase (as a donor) and fluorescent protein (as an acceptor)^12,13^. BRET results in luminescence emission from the acceptor without external excitation illumination. BRET efficiency depends on the spectral overlap between the donor emission and acceptor absorbance, and on the spatial arrangement of the donor and acceptor. High-efficient BRET has been shown to substantially increase luminescent intensity from the acceptor^14–16^. In this study, we engineered the luciferase from the luminous bacterium *Photorhabdus luminescens* and showed that the optimal fusion of a yellow fluorescent protein Venus^17^ with the luciferase significantly enhanced its brightness.

## Results

### Comparison of the gene constructs for expression of *luxA* and *luxB* in *Escherichia coli*

**Figure 1:**
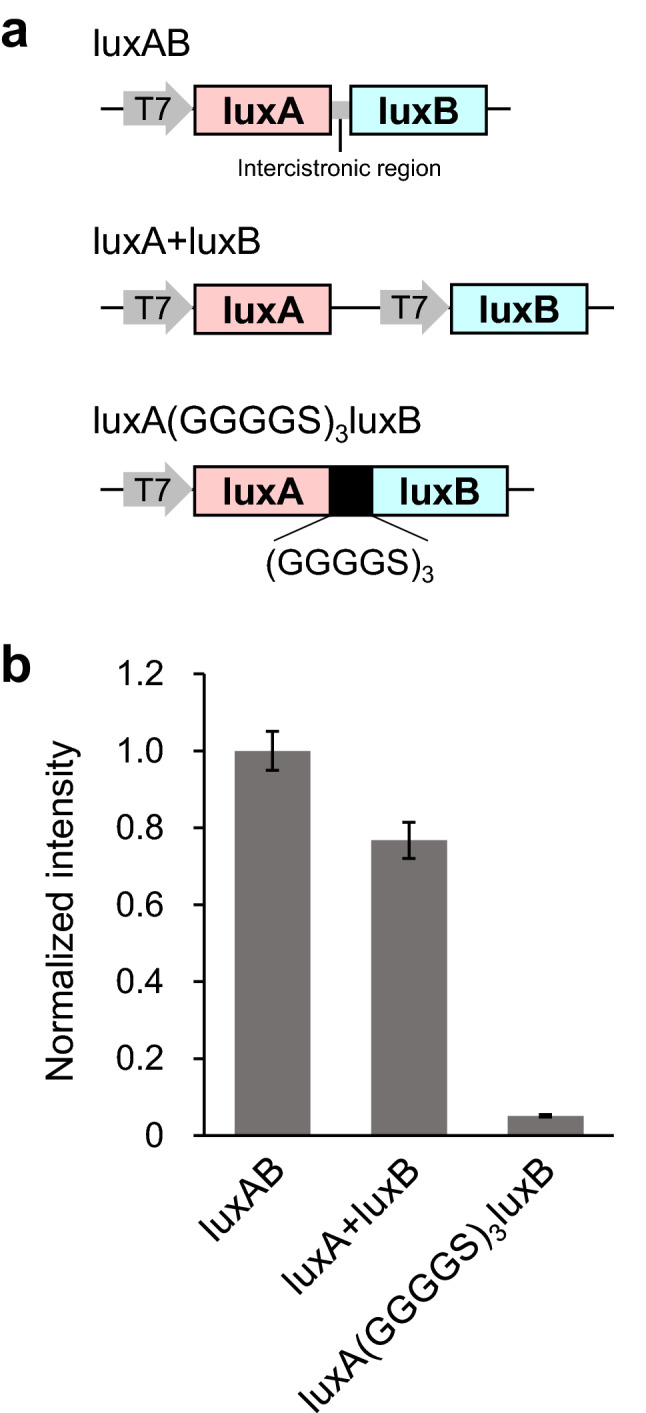
Comparison of the expression constructs of *luxA* and *luxB* genes in *E. coli*. (**a**) Schematic representation of expression constructs of *luxA* and *luxB* genes in pRSET B vector. (**b**) Luminescence intensities in the whole cell suspensions of JM109(DE3) expressing the recombinant proteins from each construct. The luminescence reaction was initiated by the addition of 1% decanal. Data are means ± SD of three different clones.

To compare the genetic constructs for expression of two subunits of the luciferase in *Escherichia coli*, we designed three constructs: bicistronic expression of *luxAB* from the original operon, dual promoter-driven expression of *luxA* and *luxB*, and fusion protein of *luxA* and *luxB* by a 15 amino acids linker (GGGGS)_3_ (Fig. 1a). The constructs were introduced into *E. coli* strain JM109(DE3), and the whole cell suspensions overexpressing the recombinant proteins were used for the measurement of bioluminescence. Luminescence reaction was induced by the addition of decanal [CH_3_(CH_2_)_8_CHO] as a luciferin. The luminescence intensity of luxA + luxB expressed by the dual promoters was not statistically significant compared to that of the bicistronic luxAB (Fig. 1b). On the other hand, the luminescence of luxA(GGGGS)_3_luxB was substantially lower (about 5% of luxAB). We decided to use the dual promoter-driven expression vector to modify the luciferase, because the luminescence intensity was equally high compared to the bicistronic luxAB but this construct had more convenient restriction enzyme sites for manipulation.

### Validation of BRET for bacterial luciferase

**Figure 2:**
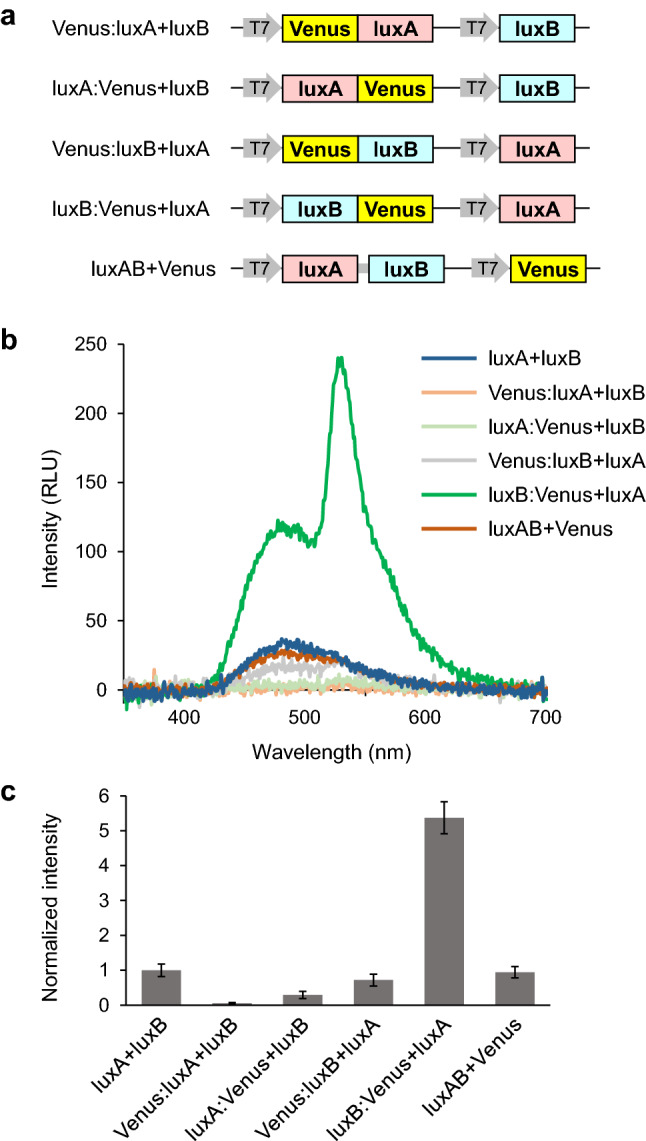
Brightness of luciferase variants fused to Venus. (**a**) Schematic representation of expression constructs in pRSET B vector. (**b**) Luminescence spectra measured in the whole cell suspensions of JM109(DE3) expressing the recombinant proteins from each construct. The luminescence reaction was initiated by the addition of 1% decanal. (**c**) Luminescence intensities of the whole cell suspensions expressing recombinant proteins. Data are means ± SD of three different clones.

We designed a chimeric protein containing Venus as a BRET acceptor. We fused Venus to N- or C-terminus of luxA or luxB (Fig. 2a). The luminescence spectrum exhibited a high BRET efficiency when Venus was fused to the C-terminus of luxB (Fig. 2b). An additional peak in the emission spectrum at around 528 nm, corresponding to the fluorescence emission maximum of Venus was identified. When Venus was expressed separately from the luciferase (luxAB + Venus), the luminescence was not significantly affected. The resulting luminescence intensity of luxB:Venus + luxA was about five times higher than luxA + luxB (Fig. 2c). Therefore, to achieve brighter luminescence, the construct design of fusing Venus on the C-terminus of luxB was used for further modifications.

We confirmed the expression levels of Venus-fused proteins by Western blot (Supplemental Fig. 1). The fusion proteins constructed in this study were expressed with an N-terminal His-tag, and detected by a His-tag antibody. The expression levels of luxA was substantially low compared to luxB in all constructs. The fusion of N- and C-terminal Venus to luxB did not affect the expression levels of their fused proteins. Therefore, the increased luminescence intensity by luxB:Venus is likely due to BRET.

### Optimization of BRET by circularly permuted Venus

**Figure 3:**
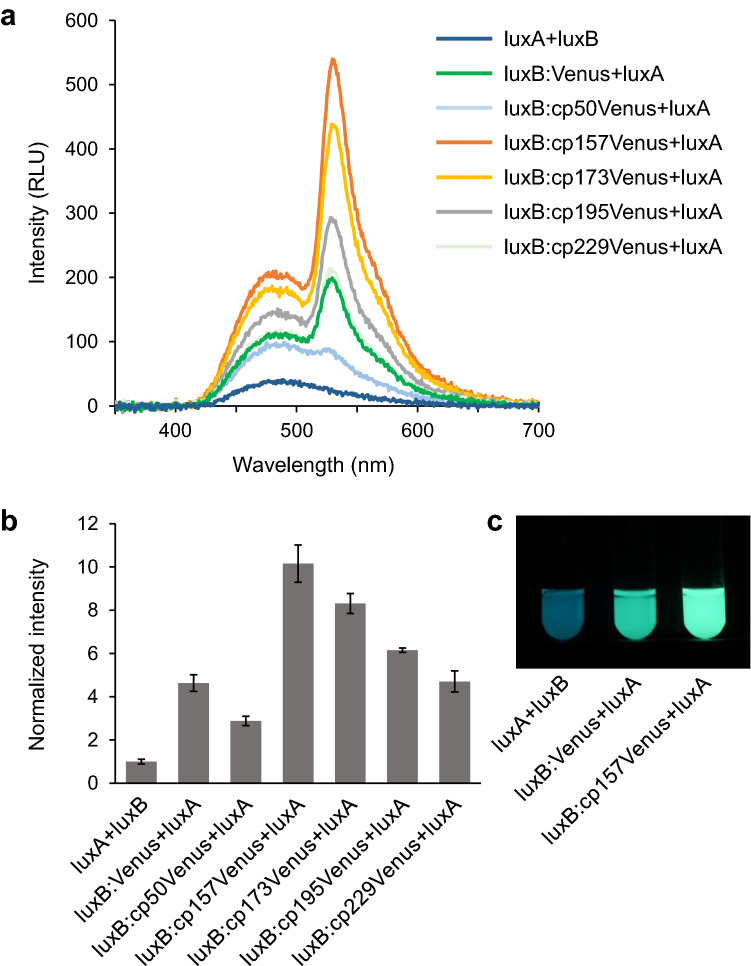
Brightness of luciferase variants fused to circularly permuted Venus. (**a**) Luminescence spectra measured in the whole cell suspensions of JM109(DE3) expressing the recombinant proteins from each construct. The luminescence reaction was initiated by the addition of 1% decanal. (**b**) Luminescence intensities of the whole cell suspensions expressing the recombinant proteins. Data are means ± SD of three different clones. (**c**) The 2 ml cultures of JM109(DE3) expressing luxA + luxB, luxB:Venus + luxA and luxB:cp157Venus + luxA after the addition of decanal. The photograph was taken by SONY α7s, ISO 5000, exposure time 2 s.

In order to further enhance the luminescence intensity, we attempted to optimize the spatial arrangement of the donor and acceptor by using circularly permuted Venus variants (cp50Venus, cp157Venus, cp173Venus, cp195Venus and cp229Venus)^18,19^. The highest BRET efficiency was observed when cp157Venus was fused to the C-terminus of luxB (Fig. 3a). The brightness of luxB:cp157Venus + luxA was about ten times higher than that of luxA + luxB (Fig. 3b). The protein expression levels of circularly permuted Venus-fused luxB, analyzed by Western blot, were unaffected by their variations (Supplemental Fig. 2). The Venus-mediated BRET had led to the color shift of light emission from blue-green to green (Fig. 3c).

### Transient expression of the engineered *lux* genes in human cells

**Figure 4:**
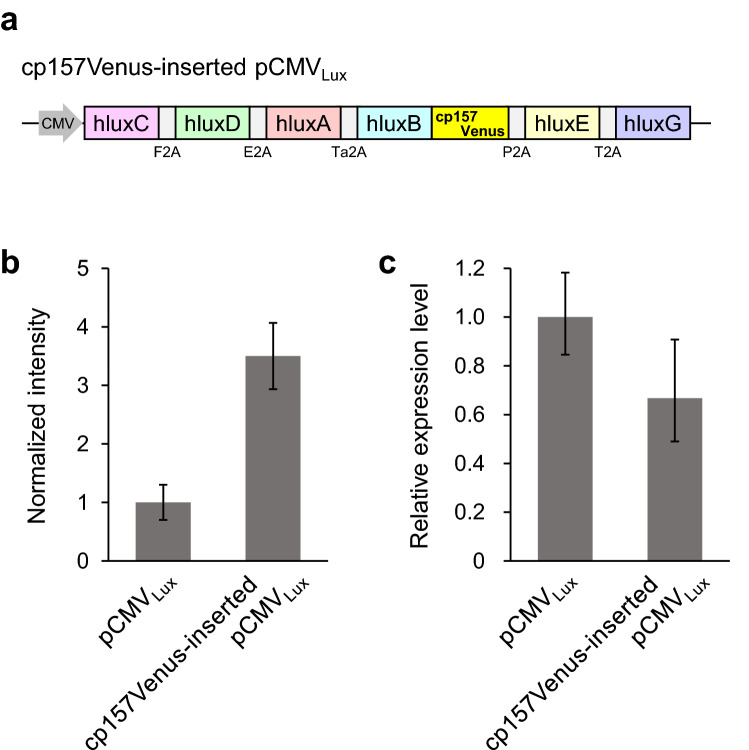
Autobioluminescence of HEK293T cells expressing pCMV_Lux_. (**a**) Schematic representation of cp157Venus-inserted pCMV_Lux_. (**b**) Luminescence intensities of HEK293T cells expressing the indicated constructs. Data are means ± SD of three different cell suspensions. (**c**) Relative quantitation of hluxB and hluxB:cp157Venus expression. The transcript level was probed with hluxB primers. Results were normalized by *GAPDH* gene expression. Values are calculated by ΔΔC_T_ from three biological replicates.

The pCMV_Lux_ vector which harbors a codon-humanized viral 2A-linked *luxCDABEG* genes has been shown to evoke autonomous bioluminescence in human cells^8^. To investigate the effect of Venus-fused luciferase on autobioluminescence intensity, we inserted cp157Venus into the C-terminus of codon-humanized luxB (hluxB) of pCMV_Lux_ (Fig. 4a), and introduced it into human embryonic kidney cell lines (HEK293T). The luminescence intensity of cells containing cp157Venus-inserted pCMV_Lux_ was about 3.5 times higher compared to conventional pCMV_Lux_ (Fig. 4b). No significant difference in gene expression levels of hluxB and hluxB:cp157Venus was detected by real-time quantitative RT-PCR analysis (Fig. 4c).

### Transient expression of the engineered *lux* genes in *Nicotiana benthamiana* leaves

**Figure 5:**
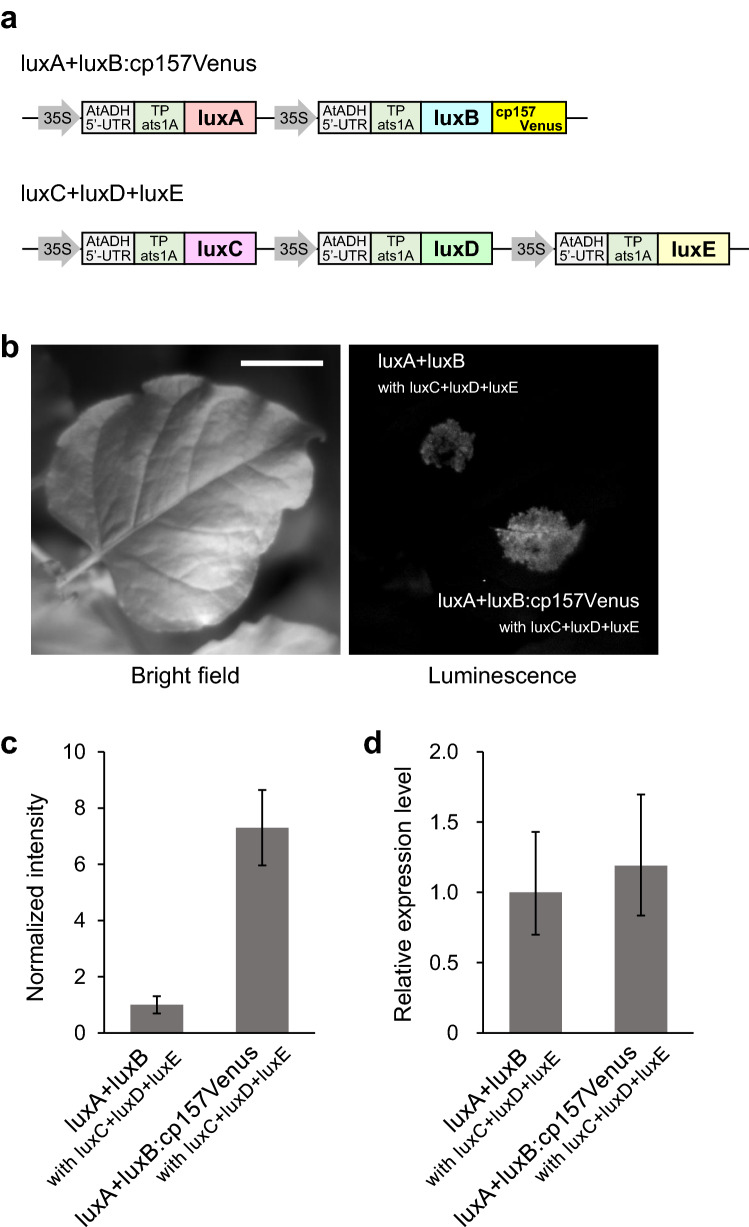
Autobioluminescence of *N. benthamiana* leaves expressing *lux* genes. (**a**) Schematic representation of expression constructs containing *lux* genes in pRI201-AN vector. (**b**) Luminescence image of the leaf cotransfected with the indicated constructs by *Agrobacterium* infiltration. The exposure time for the luminescence image was 10 s. Scale bar, 2 cm. (**c**) Luminescence intensities of leaf discs cut from the *Agrobacterium*-infiltrated regions. Data are means ± SD of three different leaves. (**d**) Relative quantitation of luxB and luxB:cp157Venus expression. The transcript level was probed with luxB primers. Results were normalized by *PP2A* gene expression. Values are calculated by ΔΔC_T_ from three biological replicates.

To expand the application of the Venus-fused luciferase to autobioluminescent plants, we constructed plant vectors expressing *lux* genes (Fig. 5a). The luxCDE complex requires fatty acid as a substrate^20^. Because fatty acid synthesis in plant is known to occur almost exclusively in the chloroplast^21^, we fused a transit peptide of *Arabidopsis thaliana* Rubisco small subunit 1A (TP*ats1A*)^22^ in front of each *lux* gene for localization of the proteins in the chloroplast. The genes were placed under the control of CaMV 35S promoter for constitutive expression. The resulting genes were integrated into two separate vectors: luxA + luxB and luxC + luxD + luxE units. *Agrobacterium tumefaciens* was transformed with each vector, and cotransfected in equal amounts into *Nicotiana benthamiana* leaves using a needle-less syringe. Autonomous bioluminescence was observed in the *Agrobacterium*-infiltrated regions (Fig. 5b). The luminescence intensity of the leaf disc expressing luxB:cp157Venus was about seven times higher compared to that expressing the non-fused luxB (Fig. 5c). No significant difference in gene expression levels of luxB and luxB:cp157Venus was observed (Fig. 5d).

## Discussion

Our results showed that the optimization of the BRET between the bacterial luciferase and yellow fluorescent protein Venus can enhance bioluminescence. The luminescence intensity relative to the protein expression level suggests that the enhanced brightness of Venus-fused luciferase was not because of the increased expression level of the luciferase, but is more likely due to BRET. BRET efficiency differed depending on the construct design, and the change in emission ratio from luciferase (490 nm) and Venus (528 nm) lead to the change in color. Among the assessed construct designs, the brightness was most enhanced when cp157Venus was fused to the C-terminus of luxB. It can be speculated that high BRET efficiency was achieved by optimizing the distance (by peptide linker) and relative dipole orientation (by circular permutation) between luciferase and cp157Venus.

The quantum yield of bacterial luciferase is about 0.1–0.16^23,24^ and Venus is about 0.6^18^. Considering that cp157Venus has a similar brightness to the wild-type Venus^18^ (therefore, similar quantum yield), maximum of six times enhancement can be expected by BRET. However, we achieved ten times enhancement by fusing luciferase to cp157Venus, which was higher than what was expected. This may have resulted from the change in functional properties of luciferase caused by the fusion of cp157Venus. For example, the firefly luciferase fused to Venus had enhanced brightness not as a result of BRET, but from other reasons that is not understood^25^. Further investigations such as 3D structure and biophysicochemical properties (e.g. *K*_*m*_) of the luxB fused with Venus may provide additional information for the cause of this additional enhancement and for further improvement of brightness.

We demonstrated that the brightness of this enhanced BRET-based luciferase was functional in autonomous luminous mammalian and plant cells generated by the coexpression of *lux* genes. Recently, autobioluminescence imaging of plant by using the fungal bioluminescence system was reported^26^. The fungal luciferase is a promising tool for autonomous luminous bioimaging, but the enzyme exhibits several drawbacks. The fungal luciferase is a temperature-sensitive enzyme, so the activity is almost lost at above 30 °C^27^. In addition, the transmembrane domain of the fungal luciferase can lead to low solubility^27^, making it difficult for high cytosolic or organelle targeted expression. The bacterial bioluminescence system would function in widely cell types including both animals^6^ and plants^7^ compared to the fungal bioluminescence system.

Naturally occurring energy transfer between bacterial luciferase and fluorescent protein has been found in some species. The *Vibrio fischeri* strain Y-1 expresses a yellow fluorescent protein YFP, and emits yellow light (around 545 nm) by energy transfer between the luciferase and YFP^28,29^. Furthermore, the blue fluorescent protein termed lumazine protein (LumP) was isolated from *Photobacterium leiognathi*, *Photobacterium phosphoreum* and *Photobacterium kishitanii*, and energy transfer between the luciferase and LumP causes blue light emission (around 475 nm)^30–32^. These YFP and LumP also enhanced the intensity of luminescence greater than three to four times^29,30^, however, the genes of YFP or LumP in these bacteria are not genetically fused to the lux genes and may not be optimal for high luminescence. Our results showed that the optimized fusion of the fluorescent protein to bacterial luciferase led to a more effective energy transfer.

Several attempts have been made to enhance the signal of bacterial bioluminescence, including codon optimization^6,33^ and random mutagenesis^11,34^. The improvement of bacterial luciferase by BRET is a useful strategy to enhance the bioluminescent signal. So far, BRET-based bacterial luciferase has been applied for the analysis of protein–protein interactions^35^, or biosensors^36^. Further optimization of BRET pair and linker in between them may improve the brightness of bacterial bioluminescence.

Luminescence using the bacterial lux system has been demonstrated in different species including plants and animals, and further development of enhanced brightness by fusing luxB to other fluorescent proteins may also be useful. For example, orange/red-light emitting luminescent proteins such as Antares^37^ and ReNL^38^ have been developed using other luciferases, which allows deep-tissue imaging by the effective light penetration of the longer wavelength. The BRET-based bacterial luciferase holds promise as a valuable autobioluminescent tool for long-term continuous imaging in live cells from bacteria to plants and animals.

## Methods

### Construction of *E. coli* expression vectors

Primers used in this study were listed in Supplementary Table 1. The *luxCDABE* genes of *Photorhabdus luminescens* was cloned from pAKlux1^39^, which was provided by Attila Karsi (Addgene plasmid #14073). For bicistronic expression of *luxAB* operon in *E. coli*, PCR-amplified *luxAB* fragment was digested with BamHI and EcoRI, and the fragment was inserted in-frame into the corresponding site of pRSET B (Thermo Fisher Scientific). For the fusion protein expression, a 15-amino-acid linker (GGGGS)_3_ was inserted between *luxA* and *luxB* by overlap extension PCR as previously described^34^. The resulting PCR product was inserted in-frame into the BamHI-EcoRI site of pRSET B.

For co-expression of two subunits of luciferase in *E. coli* by dual promoter from a single plasmid, the region including two multiple cloning sites (MCS) of pETDuet-1 (Merck) excised with BamHI and KpnI was inserted into the corresponding site of pRSET B. Venus and circularly permutated Venus series were cloned from BRAC derivatives^19^. Venus or circularly permutated Venus variant was fused to a subunit of the luciferase by an EL (glutamic acid-leucine) linker encoded by SacI recognition sequence. The fusion constructs were inserted in-frame into the BamHI-NotI site of the MCS1. The PCR-amplified fragment of another subunit of luciferase was inserted in-frame into the NdeI-KpnI site of MCS2.

### Measurement of bioluminescence in *E. coli*

The colonies of transformed *E. coli* JM109(DE3) were grown at 23 °C for 60 h in 2 ml Luria–Bertani (LB) medium to express the recombinant protein, as previously described^38^. The OD_600_ of the cultures were adjusted to 1.0 by adding LB medium. Emission spectra of the whole cell suspensions overexpressing the recombinant proteins were measured using a photonic multi-channel analyzer (PMA-12, Hamamatsu Photonics) at room temperature. Decanal is known to cross membranes^40^ and a final concentration of 1% (v/v) decanal (Wako Pure Chemical) was added just before the measurement, which is expected to be at or near saturating concentration^41^.

### Western blot

The transformed *E. coli* cells were collected and resuspended in PBS buffer with 0.2 mg/ml lysozyme. The cells were lysed by sonication, and the supernatant was collected. The samples were mixed with SDS sample buffer and boiled for 5 min. The proteins were separated by SDS-PAGE. The proteins were transferred to Immobilon-P PVDF membrane (Merck). For detection of His-tagged proteins, Anti-His-tag pAb (MBL) at a dilution of 1:5000, and the secondary anti-rabbit IgG, HRP conjugate (Promega) at a dilution of 1:5000 were used. The chemiluminescence was imaged by ECL Prime Western Blotting Detection Reagent (GE Healthcare).

### Modification of pCMV_Lux_ vector

The pCMV_Lux_ vector was purchased from 490 Biotech. A unique EcoRV site is located in the 3’ end of luxB of pCMV_Lux_, and cp157Venus was inserted into the EcoRV site of pCMV_Lux_ by In-Fusion HD cloning (Takara Bio).

### Transient expression assay in HEK293T cells

HEK293T cells (RIKEN BRC, RCB2202) were cultured to 60% confluence. Then cells were transformed with an expression vector using polyethylene imine (PEI MAX, MW 40,000, Polysciences) according to the manufacturer’s instructions, and grown for 24 h. Before measurements, transfected cells on a 4 cm culture dish were detached and suspended by 1 ml of Gibco Dulbecco’s Modified Eagle’s Medium (DMEM/F-12, 15 mM HEPES, no phenol red, Thermo Fisher Scientific). Luminescence of cells were measured by a microplate reader (SH-9000, Corona Electric).

### Construction of plant expression vectors

We prepared two types of modified pRI201-AN (Takara Bio) as follows; One pRI201-AN was digested with BamHI, and digested ends were filled in with KOD -Plus- DNA polymerase (Toyobo). The subjected DNA fragment was circularized by DNA ligase (Promega) for yielding pRI201-AN(ΔBamHI). Another pRI201-AN was digested with KpnI and EcoRI to remove the MCS2 from the vector, and self-ligation as described above for yielding pRI201-AN(ΔMCS2).

For amplification of cDNA encoding chloroplast targeting transit peptide from *A. thaliana* (TP*ats1A*, At1g67090) by RT-PCR, RNA was extracted from *A. thaliana* leaves by using ISOGEN (Nippon Gene) according to the manufacturer’s instructions. Reverse transcription was carried out using SuperScript III (Thermo Fisher Scientific) and adapter-linked oligo dT primer (Supplementary Table 1). RT-PCR was done with primer set Fwd-NdeI-TP*ats1A* and Rev-BamHI-SacI-TP*ats1A* (Supplementary Table 1), and the obtained cDNA was digested with NdeI and SacI. The fragment was ligated to the NdeI-SacI site of pRI201-AN(ΔBamHI) and pRI201-AN(ΔMCS2) for yielding pRI201-AN(ΔBamHI)-TP*ats1A* and pRI201-AN(ΔMCS2)-TP*ats1A*, respectively.

The PCR-amplified fragments of luxA, luxB, luxC, luxD and luxE were digested with BamHI and SacI. The luxA and luxC fragments were ligated to the BamHI-SacI site of pRI201-AN(ΔBamHI)-TP*ats1A* to yield pRI201-AN-TP*ats1A*:luxA and pRI201-AN-TP*ats1A*:luxC, respectively. The luxB, luxD and luxE fragments were similarly inserted into pRI201-AN(ΔMCS2)-TP*ats1A* to yield pRI201-AN-TP*ats1A*:luxB, pRI201-AN-TP*ats1A*:luxD and pRI201-AN-TP*ats1A*:luxE, respectively. The pRI201-AN-TP*ats1A*:luxB and pRI201-AN-TP*ats1A*:luxE were excised with HindIII and EcoRI and inserted into the HindIII-EcoRI site of pBluescript SK( +) (Agilent Technologies) for yielding pBS-luxB-cassette and pBS-luxE-cassette. The pBS-luxB-cassette was excised with KpnI and EcoRI and ligated to KpnI-EcoRI site of pRI201-AN-TP*ats1A*:luxA to yield pRI201-AN-TP*ats1A*:luxA + luxB. The pBS-luxE-cassette was similarly ligated to pRI201-AN-TP*ats1A*:luxC to yield pRI201-AN-TP*ats1A*:luxC + luxE. The double stranded oligonucleotide adapter which has HindIII, EcoRI and SalI sites (3RE-adapter oligonucleotide 1 and 2, Supplementary Table 1) were synthesized and ligated to KpnI-SacI site of pBluescript SK( +) to yield pBS-3RE. The pRI201-AN-TP*ats1A*:luxD was excised with HindIII and EcoRI and inserted into the HindIII-EcoRI site of the pBS-3RE for yielding pBS-luxD-cassette. The pBS-luxD-cassette was excised with KpnI and SalI and introduced into KpnI-SalI site of pRI201-AN-TP*ats1A*:luxC + luxE to yield pRI201-AN-TP*ats1A*:luxC + luxD + luxE.

The PCR-amplified fragments of luxB:cp157Venus was inserted into BamHI-SacI site of pBS-luxB-cassette by In-Fusion HD cloning to yield pBS-luxB:cp157Venus-cassete. The pBS-luxB:cp157Venus-cassette was excised with KpnI and EcoRI and ligated to KpnI-EcoRI site of pRI201-AN-TP*ats1A*:luxA to yield pRI201-AN-TP*ats1A*:luxA + luxB:cp157Venus.

### Transient expression assay in *N. benthamiana* by *Agrobacterium* infiltration

For plant material, wild-type *N. benthamiana* were germinated on soil and grown for one month at 24 °C under 24 h light. The third leaf from the top was used for *Agrobacterium* infiltration. All plant materials were handled and disposed of according to Osaka University guidelines. Transformed *A. tumefaciens* (GV3101) was collected and resuspended in infiltration buffer (10 mM MgCl_2_, 10 mM MES, pH 5.6, 100 μM acetosyringone) to OD_600_ = 0.5. The suspension was kept at room temperature for 4 h. For cotransfection of luxA + luxB and luxC + luxD + luxE, two transformants were mixed in equal amounts. A final concentration of 0.005% (v/v) silwet L-77 was added just before the infiltration. The suspension was infiltrated using a needleless syringe to the abaxial side of leaves of *N. benthamiana*. Three days after infiltration, bioluminescence was observed by a multi-functional in vivo imaging system (MIIS, Molecular Devices) equipped with EMCCD camera (iXon Ultra897, Andor Technology). For quantitative analysis of signal intensity, the infiltrated regions were cut into 5 mm diameter discs, and luminescence intensity of leaf discs were measured by a microplate reader (SH-9000).

### Quantitative analysis of gene expression

Total RNA was extracted using TRIzol reagent (Thermo Fisher Scientific) according to the manufacturer’s instructions. Before cDNA synthesis, genomic DNA contamination was removed using TURBO DNase (Thermo Fisher Scientific). One microgram of total RNA was reverse transcribed to first-strand cDNA using SuperScript III first-strand synthesis system (Thermo Fisher Scientific) with oligo(dT)_20_ primer. Real-time quantitative RT-PCR was performed on StepOne Real-Time PCR system (Thermo Fisher Scientific) with PowerUp SYBR Green Master Mix (Thermo Fisher Scientific). The baseline and threshold cycles (C_T_) for the amplification curves were calculated using the StepOne software v2.3. Results from HEK293 and *N. benthamiana* were normalized by *GAPDH* and *PP2A*^42^ gene expression, respectively. Relative level of gene expression was analyzed by the comparative C_T_ (ΔΔC_T_) method (StepOne software v2.3).

### Statistical analysis

Statistical significance was accepted at *P* < 0.05 assessed by Student’s *t*-test.

## Supplementary Information

Supplementary Information.
