# Supplementary material for: Enhanced brightness of bacterial luciferase by bioluminescence resonance energy transfer
Source: Sci Rep. 2021 Jul 22;11:14994. doi: 10.1038/s41598-021-94551-4 (PMC8298465; doi:10.1038/s41598-021-94551-4)
Supplement: Supplementary file 1 — Supplementary Information. [file 41598_2021_94551_MOESM1_ESM.pdf]

Supplementary Information

Enhanced brightness of bacterial luciferase by bioluminescence resonance energy transfer

Tomomi Kaku, Kazunori Sugiura, Tetsuyuki Entani, Kenji Osabe and Takeharu Nagai*

Author affiliation: The Institute of Scientific and Industrial Research (SANKEN), Osaka University, Ibaraki, Osaka 567-0047, Japan

*To whom correspondence should be addressed. E-mail: ng1@sanken.osaka-u.ac.jp

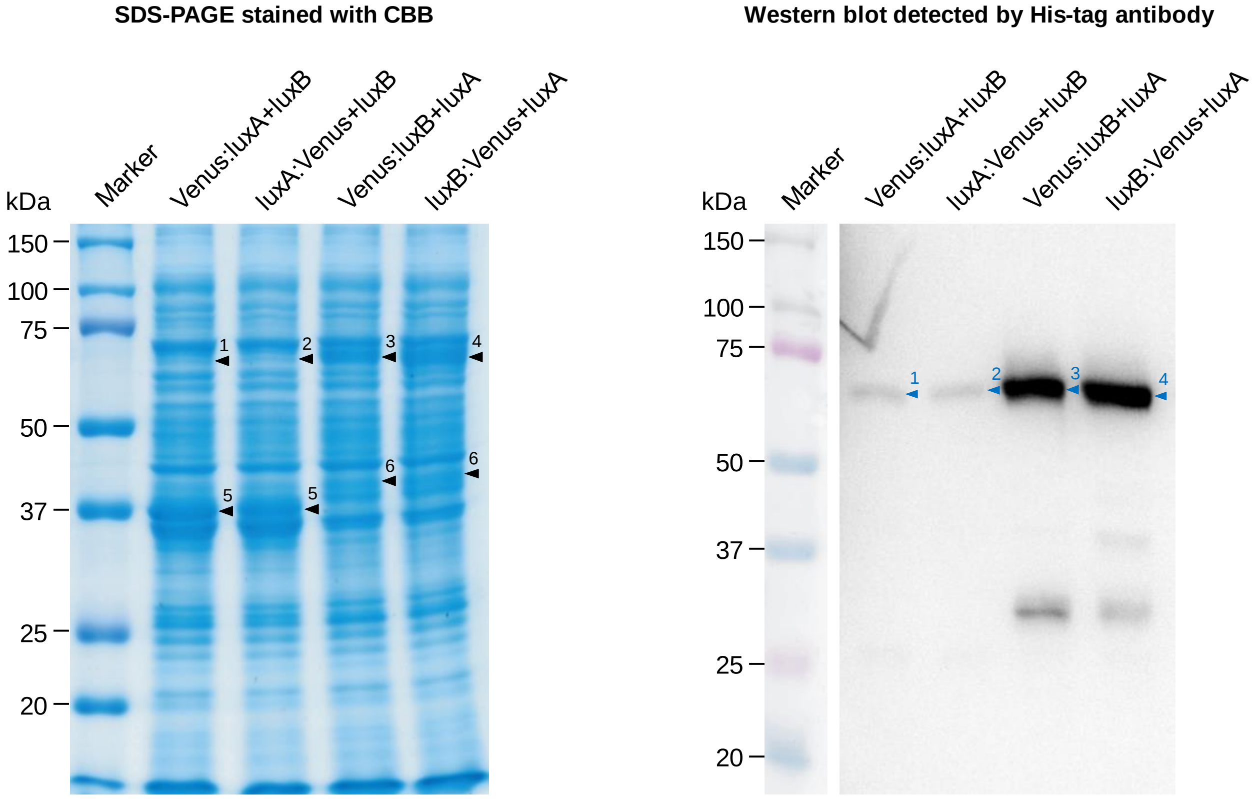

Supplementary Figure 1. Expression of lux proteins in transformed E. coli. The proteins in the soluble fractions of cell lysates were separated by SDS-PAGE, and His-tagged proteins were detected by western blot. Arrowheads show predicted positions of target proteins, and theoretical protein sizes are as follows: His_6-Venus:luxA (1) and His_6-luxA:Venus (2) are 72 kDa, His_6-Venus:luxB (3) and His_6-luxB:Venus (4) are 68 kDa, luxB (5) is 38 kDa, and luxA (6) is 41 kDa.

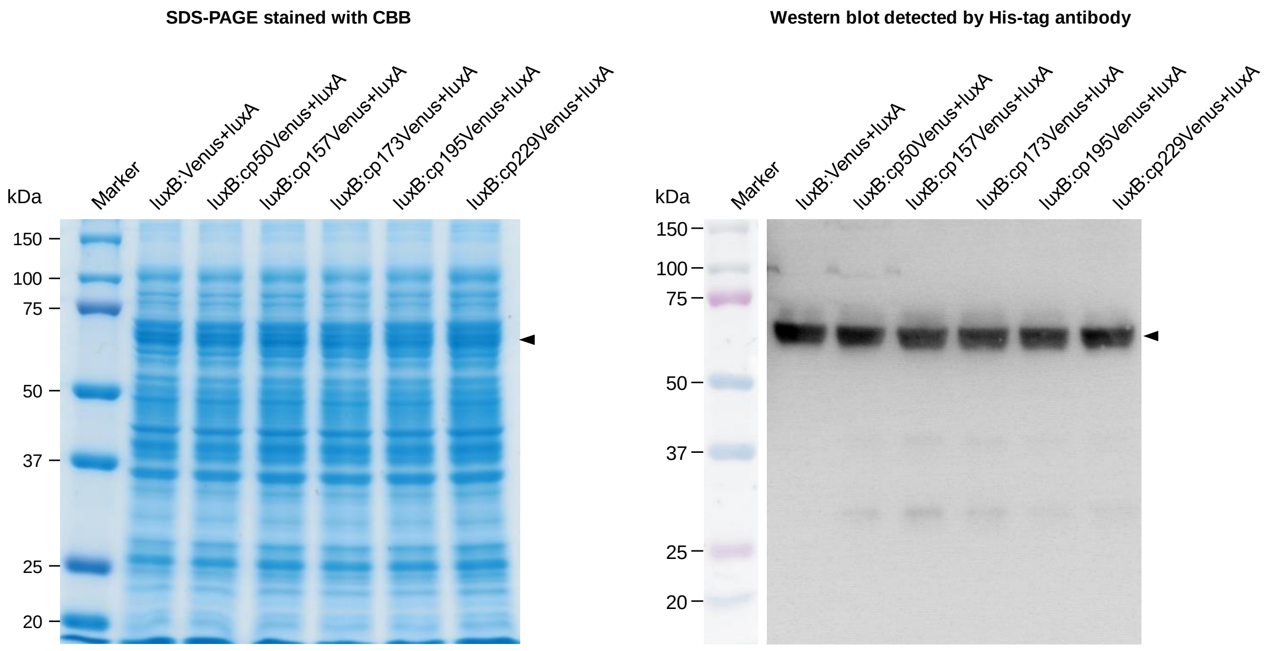

Supplementary Figure 2. Expression of luxB:cpVenus proteins in transformed *E. coli*. The proteins in the soluble fractions of cell lysates were separated by SDS-PAGE, and His-tagged proteins were detected by western blot. Arrowheads show positions of His_6-luxB:cpVenus series (69 kDa).

Supplementary Table 1. Primers used in this study.

Primer	Sequence (5' to 3')
**Primers for construction of *E. coli* expression vectors**	
Fwd-BamHI-G-luxA	CACCGGATCCGATGAAATTTGGAAACTTTTTG
Rev-EcoRI-luxB	CACCGAATTCTTAGGTATATTCCATGTGGTAC
Rev-GGGGSx3-luxA	ACTACCTCCACCTCCTGAACCTCCACCTCCACTACCTCCACCTCCATATAATAGCG<br>AACGTTG
Fwd-GGGGSx3-luxB	GGAGGTGGAGGTAGTGGAGGTGGAGGTTCAGGAGGTGGAGGTAGTATGAAATTTGG<br>ATTGTTCTTCC
Rev-NotI-luxA	CACCGCGGCCGCCTAATATAATAGCGAACGTTG
Fwd-BamHI-G-luxB	CACCGGATCCGATGAAATTTGGATTGTTCTTCC
Rev-NotI-luxB	CACCGCGGCCGCTTAGGTATATTCCATGTGGTAC
Fwd-NdeI-luxA	CACCCACCCATATGAAATTTGGAAACTTTTTG
Rev-KpnI-luxA	CACCGGTACCCTAATATAATAGCGAACGTTG
Fwd-NdeI-luxB	CACCCACCCATATGAAATTTGGATTGTTCTTCC
Rev-KpnI-luxB	CACCGGTACCTTAGGTATATTCCATGTGGTAC
Fwd-SacI-luxA	CACCGAGCTCATGAAATTTGGAAACTTTTTG
Rev-SacI-luxAΔstop	CACCGAGCTCATATAATAGCGAACGTTG
Fwd-SacI-luxB	CACCGAGCTCATGAAATTTGGATTGTTCTTCC
Rev-SacI-luxBΔstop	CACCGAGCTCGGTATATTCCATGTGGTAC
Fwd-BamHI-G-Venus	CACCGGATCCGATGGTGAGCAAGGGCGAGGAG
Rev-SacI-VenusΔstop	CACCGAGCTCCTTGTACAGCTCGTCCATG
Fwd-SacI-Venus	CACCGAGCTCATGGTGAGCAAGGGCGAGGAG
Rev-NotI-Venus	CACCGCGGCCGCTTACTTGTACAGCTCGTCC
Fwd-NdeI-Venus	CACCCACCCATATGGTGAGCAAGGGCGAGGAG
Rev-KpnI-Venus	CACCGGTACCTTACTTGTACAGCTCGTCC
Fwd-SacI-cp50Venus	CACCGAGCTCATGACCGGCAAGCTGCCCGTG
Rev-NotI-cp50Venus	CATCGCGGCCGCTTAGGTGCAGATCAGCTTC
Fwd-SacI-cp157Venus	CACCGAGCTCATGCAGAAGAACGGCATCAAG
Rev-NotI-cp157Venus	CATCGCGGCCGCTTACTTGTCGGCGGTGATA
Fwd-SacI-cp173Venus	CACCGAGCTCATGGACGGCGGCGTGCAGC
Rev-NotI-cp173Venus	CACCGCGGCCGCTTACTCGATGTTGTGGCG
Fwd-SacI-cp195Venus	CACCGAGCTCATGCTGCCCGACAACCACTAC
Rev-NotI-cp195Venus	CACCGCGGCCGCTTACAGCACGGGGCCGTCG
Fwd-SacI-cp229Venus	CACGGAGCTCATGATCACTCTCGGCATGGAC
Rev-NotI-cp229Venus	CATTGCGGCCGCTTACCCGGCGGCGGTCACG

Supplementary Table 1. Continued.

Primer	Sequence (5’ to 3’)
**Primers for modification of pCMV$_{Lux}$ vector**	
Fwd-InFusion-Ta2A-hluxB	AAATACCATATGGATCTCATGCAGAAGAACGGCATC
Rev-InFusion-cp157Venus-P2A	TGATCCCCCACCGATCTTGTCGGCGGTGATATAGAC
**Primers for construction of plant expression vectors**	
Adapter-linked oligo dT	ACTCGAATTCACGCGGCCGCATTTTTTTTTTTTTTT
Fwd-NdeI-TP*ats1A*	CCTATGCATATGGCTTCCTCTATGCTCTC
Rev-BamHI-SacI-TP*ats1A*	AATGAGCTCAATTGGATCCTCCAGATCCTCCGGAATCGGTAAGGTCAG
Fwd-BamHI-luxA	CACCGGATCCATGAAATTTGGAAACTTTTTG
Rev-SacI-luxA	CACCGAGCTCCTAATATAATAGCGAACGTTG
Fwd-BamHI-luxB	CACCGGATCCATGAAATTTGGATTGTTCTTCC
Rev-SacI-luxB	CACCGAGCTCTTAGGTATATTCCATGTGGTAC
Fwd-BamHI-luxC	TAGGATCCATGACTAAAAAAATTTCATTCATTATTAACG
Rev-SacI-luxC	TAGAGCTCATGGGACAAATACAAGGAAC
Fwd-BamHI-luxD	TAGGATCCATGGAAAATGAATCAAAATATAAAACCATC
Rev-SacI-luxD	AAGAGCTCAAGACAGAGAAATTGCTTGA
Fwd-BamHI-luxE	TAGGATCCATGACTTCATATGTTGATAAACAAG
Rev-SacI-luxE	AAGAGCTCAACTATCAAACGCTTCG
3RE-adapter oligo 1	CAAGCTTGATATCGAATTCTCGAGTCGACAGCT
3RE-adapter oligo 2	GTCGACTCGAGAATTCGATATCAAGCTTGGTAC
Fwd-InFusion-TP*ats1A*-luxB	AGGATCTGGAGGATCCATGAAATTTGGATTGTTCTTC
Rev-InFusion-cp157Venus-HSPter	CTTCATCTTCATAAGAGCTCTTACTTGTCGGCG
**Primers for real-time quantitative PCR**	
Fwd-qPCR-hluxB	TGATCTTCAAGTGGGACGACAG
Rev-qPCR-hluxB	TCGTTGTAGTTCACCAGCAC
Rev-qPCR-GAPDH	CCCTGTTGCTGTAGCCAAATTC
Rev-qPCR-GAPDH	CCCTGTTGCTGTAGCCAAATTC
Fwd-qPCR-luxB	TCGCATGCAGGAAATAACGG
Rev-qPCR-luxB	CCGAGCAGAAAACCAGAAACAG
Fwd-qPCR-PP2A	GACCCTGATGTTGATGTTCGCT
Rev-qPCR-PP2A	GAGGGATTTGAAGAGAGATTTC

Restriction enzyme sites are underlined.
